# A High-Throughput MEMS-Based Differential Scanning Calorimeter for Direct Thermal Characterization of Antibodies

**DOI:** 10.3390/bios12060422

**Published:** 2022-06-16

**Authors:** Shifeng Yu, Yongjia Wu, Shuyu Wang, Michael Siedler, Peter M. Ihnat, Dana I. Filoti, Ming Lu, Lei Zuo

**Affiliations:** 1 State Key Laboratory of Power Transmission Equipment & System Security and New Technology, Chongqing University, Chongqing 400044, China; shifengy@cqu.edu.cn; 2 Department of Mechanical Engineering, Virginia Polytechnic Institute and State University, Blacksburg, VA 24060, USA; yjwu2019@whut.edu.cn (Y.W.); wangshuyu@neuq.edu.cn (S.W.); 3 School of Civil Engineering and Architecture, Wuhan University of Technology, No. 122, Luoshi Road, Wuhan 430070, China; 4 AbbVie Deutschland, 67061 Ludwigshafen, Germany; michael.siedler@abbvie.com; 5 AbbVie Bioresearch Center, Worcester, MA 01605, USA; peter.ihnat@abbvie.com (P.M.I.); dana.filoti@abbvie.com (D.I.F.); 6 Center for Functional Nanomaterials, Brookhaven National Laboratory, Upton, NY 11079, USA; mlu@bnl.gov

**Keywords:** thermal stability, antibody, differential scanning calorimeter, MEMS

## Abstract

Calorimeters, which can be used for rapid thermal characterization of biomolecules, are getting intense attention in drug development. This paper presents a novel MEMS-based differential scanning calorimeter (DSC) for direct thermal characterization of protein samples. The DSC consisted of a pair of temperature sensors made by vanadium oxide (VO_x_) film with a temperature coefficient of resistivity of −0.025/K at 300 K, a microfluidic device with high thermal insulation (2.8 K/mW), and a Peltier heater for linear temperature scanning. The DSC exhibited high sensitivity (6.1 µV/µW), low noise (0.4 µW), high scanning rate (45 K/min), and low sample consumption volume (0.63 µL). The MEMS DSC was verified by measuring the temperature-induced denaturation of lysozyme at different pH, and then used to study the thermal stability of a monoclonal antibody (mAb), an antigen-binding fragment (Fab), and a dual variable domain immunoglobulin (DVD-Ig) at pH = 6. The results showed that lysozyme is a stable protein in the pH range of 4.0–8.0. The protein stability study revealed that the transition temperatures of the intact Fab fragment, mAb, and DVD proteins were comparable with conformational stability results obtained using conventional commercial DSC. These studies demonstrated that the MEMS DSC is an effective tool for directly understanding the thermal stability of antibodies in a high-throughput and low-cost manner compared to conventional calorimeters.

## 1. Introduction

Since the approval of the first monoclonal antibody in 1986 for treating organ rejection, monoclonal antibodies (mAb) and antibody-based therapeutics have dominated the biopharmaceutical market [1]. However, there is an increasing interest in understanding the factors that affect the stability of antibodies [2]. Thermal stability is a direct assessment of the conformational integrity of proteins [3]. Poor conformational stability can lead to tertiary structure misfolding in the variable regions, a decrease in antigen binding, and aggregation in solution [4]. Moreover, the conformational stability of mAbs and dual variable domain immunoglobulins (DVD-Igs) is directly related to their solution environments including pH value and solutes. Solution conditions impact the tertiary structures by modifying the ionization of amino acids, direct interactions, and hydration of the surface of proteins [5].

When antibodies or DVD-Igs are subjected to increasing temperature, the native tertiary state transforms to a fully denatured or unfolded state. Differential scanning calorimeters (DSCs) measure the temperature required for this transition ($T_{m}$) along with the enthalpy needed to achieve the unfolded state (ΔH). Generally, a higher $T_{m}$ indicates a more conformationally stable protein under specified conditions. As a label-free and immobilization-free method, DSC simultaneously evaluates the temperature and energy required by the protein to transform from the native to denatured state. This provides a direct understanding of the molecular interactions of samples [6,7]. Conventional commercial DSCs consume relatively large sample volumes (typically 20–300 µL) and long measurement times (~2 h) due to their fairly low power resolution and large thermal mass of the measurement systems [6]. During the DVD-Ig engineering, the precursor variable regions are selected from mAbs which demonstrate a relatively high conformational stability prescreened by DSC [8]. With the advent of automated and high-throughput cell culture processes, hundreds of mAbs and DVD-Igs can be produced in a short time, necessitating the development of a thermal analysis tool which can screen the biomolecules quickly to obtain the relative conformational differences between the potential therapeutic candidates while minimizing material consumption [9].

Standard MEMS fabrication processes have been used to develop miniaturized calorimeters to address the shortcomings of the conventional large-volume DSCs [10,11,12,13,14]. By integrating the calorimeter on the suspended Si_3_N_4_ to increase thermal insulation, a DSC can achieve a microwatt noise level using a platinum-based resistor for the temperature sensing [10]. Due to the miniaturized size, the flash DSC, a commercially available calorimeter which has been widely used for the characterization of thin-film materials, achieved a scanning rate at 1000 K/s, which is much higher compared to the rate of conventional DSCs (1 K/min) [11,15,16]. However, these micro-DSCs use an open-chamber configuration, which allows the evaporation of aqueous samples and introduces extra heat loss; therefore, they are not suitable for liquid biosample measurement. In order to overcome this drawback, a polydimethylsiloxane (PDMS)-based microfluidic device was introduced for preventing evaporation to make the DSC available for liquid sample characterization [12]. Further attempts utilized a commercially available polymeric sheet to replace the suspended Si3N4 thin film to reduce the cost and increase the reliability of the MEMS DSC [13,17]. However, evaporation of liquid still occurs when the temperature is close to the water boiling point due to the high gas permeability of PDMS [18]. There are other methods developed to enable microcalorimeters to quantitatively analyze molecules. By implementing the segmented flow technique, chip calorimeters can be used to investigate the mixing of samples with different consistencies directly [19,20]. Instead of utilizing temperature sensitivity resistors, quartz resonators based calorimeter transduced urinary creatine into temperature-dependent frequency signals [21]. An additional vacuum system can further reduce the noise of a calorimeter, thus enabling highly sensitive monitoring of metabolic activities in single cells [22].

In this paper, a MEMS DSC is introduced as a high-throughput calorimeter for the thermal characterization of antibodies. Compared to other existing DSCs, the PDMS/Flexdym double-layer microfluidic chamber is designed to hold the sample and reference materials and prevent the fluid from evaporation. Additionally, a vanadium oxide-based thermistor is developed for highly sensitive differential temperature/power measurement. A thin-film microheater is deposited under the fluidic chamber for sensitivity calibration. Linear temperature scanning is achieved by precise temperature feedback control. The MEMS DSC consumes microgram quantities of antibodies to generate an accurate thermal denaturation profile at a higher thermal scanning rate with a higher accuracy than conventional commercial instruments.

## 2. Materials and Methods

### 2.1. Materials

Lysozyme (molecular mass: 14.4 kD), mAb (144.9 kD), Fab (47.4 kD), and DVD Ig (196.4 kD) were prepared at the AbbVie Bioresearch Center (Worcester, MA). The DVD-Ig was synthesized by combining the complementarity-determining regions (CDRs) of two precursor IgG1s into one dual-targeting protein using naturally occurring peptide linkers to attach the domains to the constant framework (Figures S11 and S12). A template was created from the variable domain DNA of the two precursor mAbs and cloned into an appropriate expression vector. The mAb and DVD-Ig proteins were expressed in Chinese hamster ovary (CHO) cells and grown in bioreactors. After an appropriate incubation period, the CHO cells were harvested and lysed. The supernatant was purified by protein A chromatography and anion filtration, and then diafiltered into a stabilizing buffer. The corresponding Fabs were prepared by digesting the mAbs with papain and purifying by size-exclusion chromatography (SEC). The variable domains of the DVD-Ig, mAb, and Fab are antigens specific for tetanus toxoid. The lysozyme was dialyzed to a concentration of 10 mg/mL against different buffers (50 mM NaCl and 50 mM sodium acetate at pH = 4.0, 50 mM NaCl and 50 mM sodium phosphate at pH = 6.0, and 50 mM NaCl and 50 mM sodium phosphate at pH = 8.0) to study the pH effect on its thermal stability. The mAb, Fab region, and DVD-Ig were dialyzed against the same buffer containing 15 mM histidine at pH = 6 with the same concentration of 5 mg/mL.

### 2.2. Design of the MEMS-Based DSC

**Figure 1:**
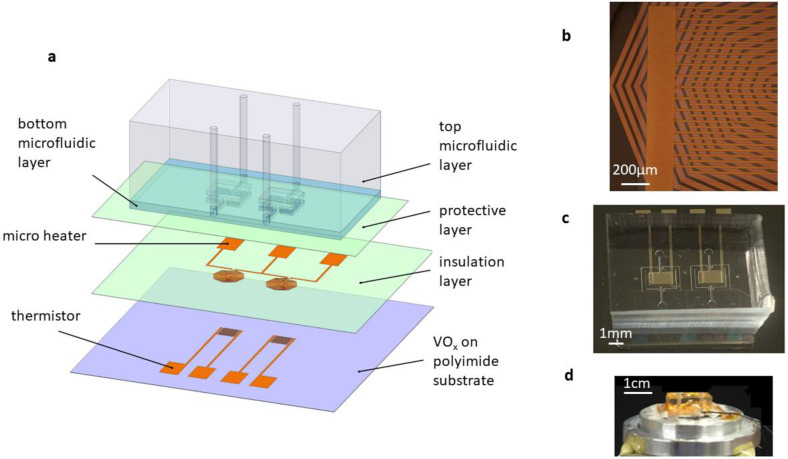
The MEMS DSC for the protein stability study. (**a**) Schematic diagram showing the components of the MEMS DSC. (**b**) Microstructure of the microheater and thermistor. (**c**) Prototype of the integrated MEMS DSC. (**d**) MEMS DSC placed on the heating stage for linear temperature scanning.

The MEMS-based DSC consisted of a pair of PDMS/Flexdym double-layer microfluidic chambers, two microheaters, two thermistors, and several polyimide protective layers (Figure 1a–d). The details of the fabrication processes are illustrated in the Supplementary Materials (Figure S1). There were two identical Flexdym-based microfluidic chambers with an identical volume of 0.63 µL, which held materials for the calorimetric measurements. These chambers were connected to the inlet and outlet ports by microfluidic channels situated on a freestanding polyimide substrate. In the experiment, liquid sample was introduced into the chamber through the inlet channel using a syringe. The polyimide diaphragm, along with air cavities surrounding the chambers, provided high thermal insulation that enabled sensitive calorimetric measurements. There were several function layers in the MEMS DSC. From the bottom to the top, there were a polyimide substrate layer, a thin-film VO_x_-based thermistor layer located underneath the centers of the calorimetric chambers, a polyimide-based dielectric layer, a thin-film resistive microheater layer under each chamber center, and a polyimide-based layer for packaging. The microheaters were designed to generate a constant differential power for calorimetric calibration. The VO_x_-based thermistors were designed for differential temperature sensing due to their high TCR and ease of fabrication. In particular, polyimide was chosen as the diaphragm material due to its excellent mechanical stiffness (Young’s modulus of 2.5 GPa), thermal stability (glass transition temperature of 285 °C), and low thermal conductivity (0.12 W/m⋅K) [23]. The double-layer design of the microfluidic device was aimed at improving the thermal insulation performance of the MEMS DSC while minimizing evaporation during temperature scanning. The detailed design and thermal analysis of the double layer microfluidic chamber can be found in the Supplementary Materials (Figures S2–S4). In particular, Flexdym was used to fabricate the bottom microfluidic layer because of its low thermal conductivity (0.27 W/(m⋅K)), low gas permeability, and good biocompatibility [24,25]. PDMS was used to fabricate the top microfluidic layer with air cavities embedded to improve the thermal insulation of the DSC device.

### 2.3. Working Principle of the MEMS-Based DSC

In DSC measurement, heat fluxes were added to the reference and sample chambers through Peltier heating to achieve linear temperature scanning. For both the reference and the sample chambers, part of the heat was lost to the surrounding environment by heat convection, conduction, and radiation. The energy conservation equations for the reference and sample chambers are given by Equations (1) and (2) [26], respectively.

(1) $$C_{r}\frac{dT_{r}}{dt}+G_{r}\left(T_{r}-T_{0}\right)=P_{0}\left(t\right),$$

(2) $$C_{s}\frac{dT_{s}}{dt}+G_{s}\left(T_{s}-T_{0}\right)=P_{0}\left(t\right)+P_{diff},$$

where $\;P_{0}\left(t\right)$ is the heat flux added to the sample and reference through Peltier heating, $P_{diff}$ is the differential heat flux between the sample and reference recorded by the thermistors during the scanning test, $C_{s}$ and $C_{r}$ are the effective heat capacity of the sample and the reference, respectively, $G_{s}$ and $G_{r}$ are the effective heat convection coefficients of the sample and reference chambers, respectively, $T_{s}$ and $T_{r}$ are the real time temperatures of the sample and reference chambers, respectively, and $T_{0}$ is the environment temperature.

Considering that the sample and reference chambers were identical, $C_{s}=C_{r}=C$ was assumed in the analysis when the chambers were filled with buffer. Furthermore, it was assumed that the reference and sample chambers were exposed to the same thermal environment; thus, $G_{s}=G_{r}=G$. Subtracting Equation (2) from Equation (1) gives

(3) $$\frac{d\Delta T}{dt}+\frac{G}{C}\Delta T-\frac{P_{diff}}{C}=0,$$

where ΔT is the temperature difference between the reference and sample chambers. Assuming that $\Delta T=u\left(t\right)e^{\left(-\frac{G}{C}\right)t}$ is the solution of Equation (3), then it can be substituted into Equation (3) to solve for u(t).

(4) $$u=\int \frac{P_{diff}}{C}e^{\left(\frac{G}{C}\right)t}dt.$$

Then,

(5) $$\Delta T=\int \frac{P_{diff}}{C}e^{\left(\frac{G}{C}\right)t}dte^{\left(-\frac{G}{C}\right)t}.$$

If $P_{diff}$ is a constant, for example, a step function, then Equation (5) can be simplified as

(6) $$\Delta T=\frac{P_{diff}}{G}=HP_{diff},$$

where *H* is the effective thermal resistance of the system.

In the MEMS-based DSC, the two thermistors located under the center of the sample and reference chambers, together with two external decade resistor boxes, formed a Wheatstone bridge to detect the temperature difference induced by the state change of the sample. The temperature difference could be further converted to $P_{diff}$ and finally converted to the heat capacity and enthalpy change. The thermistors were fabricated on vanadium oxide thin film with a high temperature coefficient of resistance (TCR) [27].

(7) $$V_{out}=\frac{1}{4}V_{in}\alpha HP_{diff},$$

where $V_{out}$ is the output voltage of the Wheatstone bridge, $V_{in}$ is the input voltage of the bridge fixed at 1 V, ΔT is the temperature difference between the sample and reference area, and α is the temperature coefficient of resistance (TCR) of the thermistor. The TCR measurement of the thermistor can be found in the Supplementary Materials (Figure S6). The common mode rejection of the Wheatstone bridge could significantly reduce the noise such as room temperature fluctuations in the sample and reference regions.

The sensitivity of the DSC, which was evaluated by the output voltage over the input differential power, can be expressed by Equation (8).

(8) $$S=\frac{V_{out}}{P_{diff}}=\frac{1}{4}V_{in}\alpha H.$$

In the DSC measurement, $P_{diff}$ was induced by the denaturation of the sample material. During the denaturation process, the heat capacity of the sample changed dramatically. The relationship between the heat capacity change ($\Delta C_{p})$ and $P_{diff}$ is expressed by Equation (9).

(9) $$\Delta C_{p}=\frac{P_{diff}}{\beta }.$$

Combining Equations (8) and (9), $\Delta C_{p}$ can be directly expressed with the output voltage $V_{out}$.

(10) $$\Delta C_{p}=\frac{V_{out}}{S\beta },$$

where β is the scanning rate. The heat capacity can be further normalized and then integrated to obtain the enthalpy change (ΔH) of the molecule during denaturation expressed by Equation (11).

(11) $$\Delta H=\int \Delta C_{p}dT.$$

## 3. Results

### 3.1. DSC Measurements

#### 3.1.1. Experimental Setup

The DSC measurement system is shown in Figure S5. The calorimeter chip was connected to the lock-in amplifier for differential signal recording. A customized chamber with a transparent cover was used to reduce signal fluctuations due to thermal disturbances from the surroundings. Linear temperature scanning was added to the calorimeter through a TE precise temperature controller powered by a Keitheley 2200 power supply. The overall DSC system was controlled by a LabView program. Before experiments, the VOx thermistors and the microheaters were treated by annealing to increase their stability and then calibrated with a high-temperature probe station (AO 600 Compact Rapid Thermal Annealing System, MBE). The calibration results are shown in Figures S6 and S7. The temperature fluctuation of the DSC was within 40 μK when it reached equilibrium (Figure S8).

#### 3.1.2. Device Characterization

**Figure 2:**
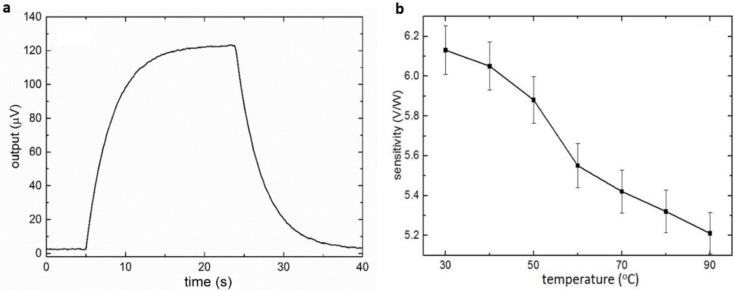
The sensitivity characterization of the MEMS DSC. (**a**) Step response of the MEMS-based DSC (20 µW input, 30 °C). (**b**) Relationship between the sensitivity and the temperature (error bars represent the stand errors of the sensitivity).

In each characterization, five devices were used to evaluate the consistency of the MEMS DSC. The sensitivity and time constant were measured by the thermal response tests. In the thermal response test, the sample and reference chambers were filled with 15 mM histidine buffer (pH = 6). A constant power was added to the microheater under the sample chamber for a certain amount of time. The power generated a temperature difference, resulting in a resistance difference between the thermistors under the sample and reference chambers and causing the unbalance of the Wheatstone bridge. The sensitivity of the DSC system was obtained by dividing the output voltage by the power applied. As a first-order system, the time constant could be derived directly from the step response. During the characterization process, different input powers were loaded to the DSC, and the voltage output was consistent (Figure S9). Figure 2a shows the thermal response of the DSC system when 20 µW was loaded at 5 s and removed at 24 s. By fitting the rising curve to the first-order system, the time constant was 3.3 s. The sensitivity of the DSC was 6.1 V/W at 30 °C. The relationship between the sensitivity and the temperature was further studied by repeating the experiment at different temperature points for three times (Figure 2b). The error bars represent the standard error of the sensitivity. The sensitivity slightly decreased as the temperature increased. According to Equation (8), the sensitivity is determined by the input voltage of the Wheatstone bridge, the TCR of the thermistor, and the thermal resistance of the DSC system. As a semiconductor material, the TCR of the VO_x_ was reduced as the temperature increased (Figure S5), which explained the sensitivity decrease with temperature.

**Figure 3:**
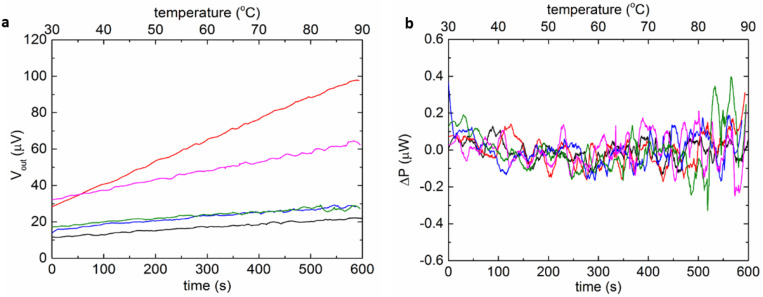
The baseline characterization of the MEMS DSC. (**a**) The raw baselines of the MEMS-based DSC (repeated five times). (**b**) The fluctuation of the baselines upon removing the linear parts.

The baseline repeatability of the MEMS DSC was characterized when both the sample and reference chambers were filled with the same buffer containing 15 mM histidine with the pH = 6 (Figure 3). While the scanning rate of the MEMS DSC in measurement was in the range of 5 to 45 °C/min, 5 °C/min was applied during baseline characterization to allow the extraction of more data points. The linear drift was mainly caused by the asymmetry of the two chambers [28]. The largest slope of the output drift was 0.6 µV/s. The sensitivity of the MEMS DSC was around 6 µV/µW, and the fluctuation level was 0.4 µW. The fluctuation of the baseline was the main source of noise of the system, which determined the detection limit of the MEMS DSC.

#### 3.1.3. pH Effect on the Thermal Stability of Lysozyme

The lysozyme measurements were carried out using the MEMS-based calorimeter at the upper limit of the scanning rate (45 °C/min) to increase the resolution of heat capacity measurement, within a temperature range from 30 °C to 100 °C. The incubation time was 2 min before scan, and the equilibrium time (post scan) was about 5 min, suggesting that the total time per measurement was about 8.5 min. The heat capacity thermograms were obtained by subtracting the baseline and interpolating a cubic baseline in the transition region. Then, the thermograms were normalized to the molar concentration of each protein and further integrated to obtain the enthalpy change. The peaks in the thermograms obtained by the experiments were the transition temperatures of the lysozyme under different pH values. A repeatability study was also carried out during the lysozyme test (Figure S10), suggesting the suitability of the device for disposable use.

**Figure 4:**
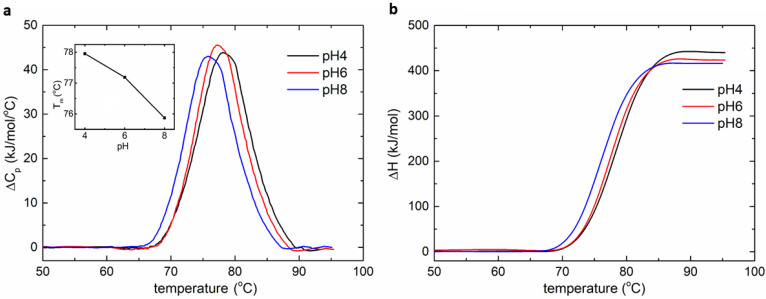
The DSC measurement of lysozyme sample. (**a**) The normalized heat capacity change as a function of temperature. The inset shows the relationship between the transition temperature and pH. (**b**) The normalized enthalpy change as a function of temperature.

Figure 4 shows the normalized heat capacity change and enthalpy change of lysozyme over the scanning temperature at different pH values. The experiment results, such as the transition temperature and enthalpy change, are consistent with the literature [29,30], demonstrating the functionality of the MEMS DSC in the protein study. In the pH interval of 4–8, the transition temperature of lysozyme ranged from 78.0 °C to 75.8 °C, while the normalized enthalpy change varied from 440 kJ/mol to 416 kJ/mol. The results showed that lysozyme is active in the pH interval of 4–8.

#### 3.1.4. Thermal Stability of the mAb, Fab, and DVD-Ig

**Figure 5:**
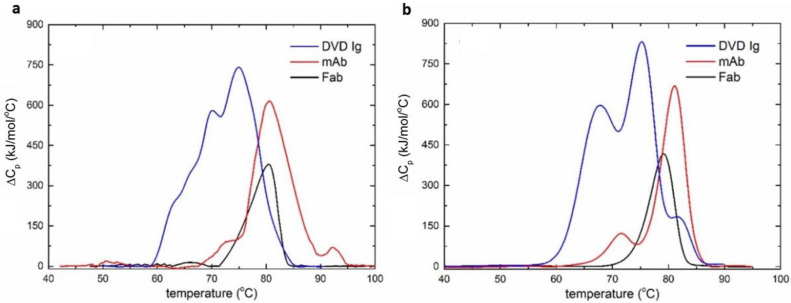
The DSC curves of the three proteins. (**a**) The DSC curves of the three proteins obtained by the MEMS-based DSC. (**b**) The DSC curves of the three proteins obtained by the MicroCal VP-Capillary DSC.

The temperature-induced denaturation of the mAb, Fab, and DVD-Ig was measured using the MEMS DSC at a scanning rate of 45 °C/min with temperature increasing from 30 °C to 100 °C (Figure 5a). To verify the performance of the MEMS-based DSC, the thermal stability profiles of the three proteins were measured using the commercial MicroCal VP-capillary DSC (Malvern/MicroCal, Northampton, MA) (Figure 5b). MicroCal VP-capillary DSC is a benchtop-sized analytical tool that is widely used in the pharmaceutical industry. Compared to the MEMS DSC that utilizes a top-down structure with a microfluidic chamber to contain the material and a heating module underneath, the capillary DSC uses a U-shape capillary to hold the sample with the surrounding heater for the temperature scan. In each measurement, a sample volume of 500 µL at 1 mg/mL was analyzed at a scanning speed of 1 °C/min with temperature increasing from 30 °C to 100 °C. It took about 80 min to finish one run, which was much longer compared to the MEMS DSC (8.5 min). The transition temperatures and calorimetric transition enthalpies were obtained by deconvolution of the thermal profiles measured by the VP-capillary DSC and MEMS DSC, respectively.

## 4. Discussion

The performance of the MEMS-based DSC was compared with the commercial DSC. The transition temperatures and enthalpy changes are parameters used to evaluate the conformational stability of the antibodies in solution [31]. ΔH is proportional to the endothermic energy required to unfold the corresponding domain of the protein. The transition temperatures and relative calorimetric areas of the deconvoluted transitions were compared between both devices for three proteins.

The thermal denaturation profiles of the Fab obtained by both the MEMS and the commercial calorimeter showed one transition at approximately 80 °C (Table 1). As expected, the denaturation profiles of the mAb obtained by both the DSC and the MEMS units demonstrated three transitions. The first and second transition temperatures measured by both devices matched quite well with a temperature difference less than 2 °C. However, the third transition temperature for the unfolding profile of the mAb obtained by the MEMS unit was more than 10 °C higher than that measured by the commercial calorimeters. Moreover, the relative area of the second transition in the denaturation profile was overestimated by the MEMS calorimeter. The more complex DVD-Ig was deconvoluted to reveal four thermal transitions when using both devices [32]. The transition temperatures obtained by both devices were consistent with a difference less than 5 °C at each point (Table 1). The commercial DSC measurements showed that the first two transitions were likely correlated with the denaturation processes of the outer and inner domains of the bispecific Fab, which required the most energy to unfold. The relative calorimetric areas from the MEMS calorimeter, however, showed that the second and third transitions occupied the largest areas.

**Figure 6:**
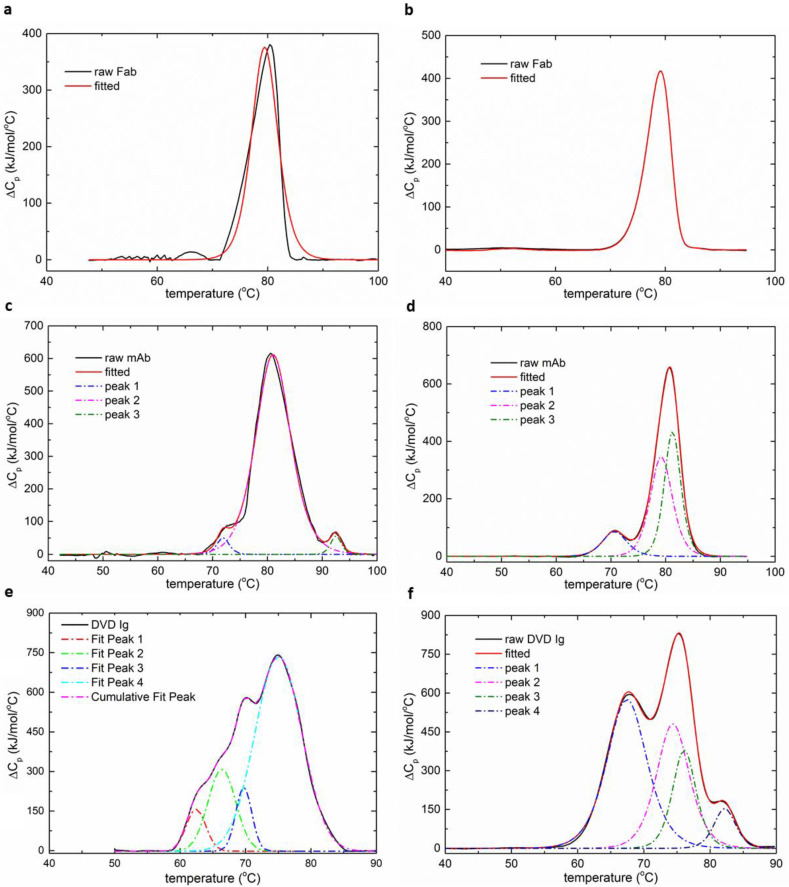
The deconvolution of the normalized DSC curves for the three protein samples. (**a**,**c**,**e**) Heat capacity diagrams measured by the MEMS DSC. (**b**,**d**,**f**) Heat capacity diagrams measured by the MicroCal VP-DSC.

The thermal denaturation profiles of immunoglobulins and other proteins reflect the transitions associated with the individual subunits. The temperatures and enthalpies of unfolding shed light on the conformational stabilities of these individual domains. Domains that have more complexity and internal peptide contacts generally require more energy to unfold and preserve larger enthalpies. The Fab domains of the bispecific and monoclonal antibody, which contain the variable or antigen-binding region, required more energy for unfolding when compared with the CH_2_ or CH_3_ regions (Figure 6). Moreover, the profiles show the interactions between domains during unfolding or cooperativity. Differences in thermal denaturation profiles are caused by variation in primary amino-acid sequences between largely similar proteins and differences in solution conditions, such as pH or excipients for the same protein. As a result, it is more suitable to measure the transition temperatures of the proteins rather than enthalpies or interactions in different domains when using the MEMS calorimeter. Compared to existing microcalorimeter devices, the MEMS DSC is not only highly sensitive and efficient, but also the first to enable the characterization of antibodies with multiple thermal domains (Table 2).

## 5. Conclusions

This paper presented an MEMS-based differential scanning calorimeter (DSC) and demonstrated the application of the MEMS-based calorimeter to obtain the thermal denaturation profiles of antibodies such as lysozyme, mAb antibody, its corresponding Fab fragment, and the mAb-based DVD-Ig. The key conclusions of this paper are summarized as follows:

1. The MEMS calorimeter achieved the purpose of screening large numbers of therapeutic proteins for conformational stability on the basis of transition temperature at a high throughput. This is the first time that an MEMS-based DSC has been used to study the interactions between different domains, which affect the thermal stability of antibodies during unfolding.
2. The denaturation profiles measured by the MEMS-based DSC were compared with those obtained using a commercial DSC device. The comparison results verified the capability of the MEMS-based DSC in the thermal characterization of complex protein samples. Its accuracy in measuring the relative enthalpy of domain unfolding following deconvolution of the profiles can be further improved in future work. This can be achieved by improving the thermal insulation of the fluid chamber and enhancing the sensitivity of the device.
3. The MEMS-based DSC had a much higher thermal profile screening throughput than the commercial DSC, which can significantly reduce the time and cost for the development of a new drug. Furthermore, the sample consumption was 0.63 µL, which was about 800 times smaller compared to the commercial DSC. The micro size of the sample indicated a large surface-to-volume ratio, thus enabling efficient thermal management and a higher scanning rate compared to the commercial DSC. The MEMS-based DSC achieved a high scanning rate of 45 °C/min with a minimum sample consumption of 0.63 µL. The time consumption of each run for the MEMS DSC was 8.5 min, which is 10 times shorter than the commercial DSC, while the sample consumption was 500 times smaller than the commercial DSC.

## Figures and Tables

**Table 1:** Comparison of the DSC curves for denaturation of the three proteins measured by the VP DSC and MEMS DSC.

Sample	Measuring Device	Transition 1		Transition 2		Transition 3		Transition 4	
		*T_m_*_1_ (°C)	∆*H*_1_ (%)	*T_m_*_2_ (°C)	∆*H*_2_ (%)	*T_m_*_3_ (°C)	∆H_3_ (%)	T_m4_ (°C)	∆H_4_ (%)
Fab	VP DSC	78.8	100%	-	-	-	-	-	-
	MEMS DSC	79.5	100%	-	-	-	-	-	-
mAb	VP DSC	70.7	12%	79.2	45%	81.3	43%	-	-
	MEMS DSC	71.9	3%	81.1	95%	92.5	3%	-	-
DVD-Ig	VP DSC	67.5	45%	74.4	31%	76.1	18%	82.2	7%
	MEMS	64.3	16%	69.7	29%	74.7	34%	78.0	21%

**Table 2:** Comparison of the MEMS DSC and other existing DSCs.

Scanning Rate °(C/min)	Temperature Sensing Method	Sensitivity (μV/μW)	Noise Level (μW)	Sample Volume (μL)	Target Materials	References
1	Thermocouple	-	0.1	370	Proteins and other biomolecules	MicroCal VP capillary DSC
20	Germanium thermistor	-	0.001	0.1	Enzyme (glucose oxidase)	Urban group [33]
5	Sb–Bi thermopile	4.78	0.021	4.78	Protein (lysozyme)	Lin group [13]
5–45	Vanadium oxide thermistor	6.1	0.4	0.63	Proteins and complex antibodies	This work

## Supplementary Materials

The following supporting information can be downloaded at: https://www.mdpi.com/article/10.3390/bios12060422/s1, Figure S1: The fabrication process of the MEMS DSC device; Figure S2: The structure of the microfluidic device; Figure S3: Three microfluidic device designs and the FEA simulation results of their step response; Figure S4: Step response of the three microfluidic device designs; Figure S5: The schematic diagram of the MEMS DSC measurement system; Figure S6: Calibration of the VO_x_-based thermistor; Figure S7: Heating calibration of the MEMS DSC; Figure S8: Temperature fluctuation of the MEMS DSC when in equilibrium; Figure S9: (a) Step response of the MEMS DSC to different input powers. (b) Linear fit of the output voltage to the input power; Figure S10: Repeatability test of the DSC device; Figure S11: The molecular structure of the Fab, mAb, and DVD Ig; Figure S12: Surface charge distribution of the Fab, mAb, and DVD Ig. The surface electrostatic potential was calculated using the PyMOL molecular graphics program (white, neutral; blue, positive charge; red, negative charge).

## References

[1] Ecker D.M., Jones S.D., Levine H.L. (2015). The therapeutic monoclonal antibody market. MAbs.

[2] Leavy O. (2010). Therapeutic antibodies: Past, present and future. Nat. Rev. Immunol..

[3] Ewert S., Honegger A., Pluckthun A. (2004). Stability improvement of antibodies for extracellular and intracellular applications: CDR grafting to stable frameworks and structure-based framework engineering. Methods.

[4] Chiti F., Dobson C.M. (2006). Protein misfolding, functional amyloid, and human disease. Annu. Rev. Biochem..

[5] Wang W., Singh S., Zeng D.L., King K., Nema S. (2007). Antibody structure, instability, and formulation. J. Pharm. Sci..

[6] Wen J., Arthur K., Chemmalil L., Muzammil S., Gabrielson J., Jiang Y. (2012). Applications of differential scanning calorimetry for thermal stability analysis of proteins: Qualification of DSC. J. Pharm. Sci..

[7] Bruylants G., Wouters J., Michaux C. (2005). Differential scanning calorimetry in life science: Thermodynamics, stability, molecular recognition and application in drug design. Curr. Med. Chem..

[8] Rothlisberger D., Honegger A., Pluckthun A. (2005). Domain interactions in the Fab fragment: A comparative evaluation of the single-chain Fv and Fab format engineered with variable domains of different stability. J. Mol. Biol..

[9] Burton L., Gandhi R., Duke G., Paborji M. (2007). Use of microcalorimetry and its correlation with size exclusion chromatography for rapid screening of the physical stability of large pharmaceutical proteins in solution. Pharm. Dev. Technol..

[10] Carreto-Vazquez V.H., Wójcik A., Liu Y.-S., Bukur D., Mannan M. (2010). Miniaturized calorimeter for thermal screening of energetic materials. Microelectron. J..

[11] Mathot V., Pyda M., Pijpers T., Poel G.V., van de Kerkhof E., van Herwaarden S., van Herwaarden F., Leenaers A. (2011). The Flash DSC 1, a power compensation twin-type, chip-based fast scanning calorimeter (FSC): First findings on polymers. Thermochim. Acta.

[12] Wang B., Lin Q. (2013). Temperature-modulated differential scanning calorimetry in a MEMS device. Sens. Actuators B-Chem..

[13] Jia Y., Wang B., Zhang Z., Lin Q. (2015). A polymer-based MEMS differential scanning calorimeter. Sens. Actuators A-Phys..

[14] Wang S.Y., Yu S., Siedler M., Ihnat P.M., Filoti D.I., Lu M., Zuo L. (2018). A power compensated differential scanning calorimeter for protein stability characterization. Sens. Actuators B-Chem..

[15] Van Herwaarden S., Iervolino E., Van Herwaarden F., Wijffels T., Leenaers A., Mathot V. (2011). Design, performance and analysis of thermal lag of the UFS1 twin-calorimeter chip for fast scanning calorimetry using the Mettler-Toledo Flash DSC 1. Thermochim. Acta.

[16] Iervolino E., van Herwaarden A.W., van Herwaarden F.G., van de Kerkhof E., van Grinsven P., Leenaers A., Mathot V., Sarro P. (2011). Temperature calibration and electrical characterization of the differential scanning calorimeter chip UFS1 for the Mettler-Toledo Flash DSC 1. Thermochim. Acta.

[17] Jia Y., Su C., He M., Liu K., Sun H., Lin Q. (2019). Isothermal titration calorimetry in a 3D-printed microdevice. Biomed. Microdev..

[18] Trung N.B., Saito M., Takabayashi H., Viet P.H., Tamiya E., Takamura Y. (2010). Multi-chamber PCR chip with simple liquid introduction utilizing the gas permeability of polydimethylsiloxane. Sens. Actuators B-Chem..

[19] Lerchner J., Sartori M.R., Volpe P.O., Förster S., Mazik M., Vercesi A.E., Mertens F. (2021). Segment fusion chip calorimetry: A new method for the investigation of fast reactions. J. Therm. Anal. Calorim..

[20] Lerchner J., David K.A., Unger F.T., Lemke K., Förster T., Mertens F. (2017). Continuous monitoring of drug effects on complex biological samples by segmented flow chip calorimetry. J. Therm. Anal. Calorim..

[21] Gaddes D., Reeves W.B., Tadigadapa S. (2017). Calorimetric Biosensing System for Quantification of Urinary Creatinine. ACS Sens..

[22] Hong S., Dechaumphai E., Green C.R., Lal R., Murphy A.N., Metallo C.M., Chen R. (2020). Sub-nanowatt microfluidic single-cell calorimetry. Nat. Commun..

[23] Arevalo A., Byas E., Conchouso D., Castro D., Ilyas S., Foulds I.G. A Versatile Multi-User Polyimide Surface Micromachinning Process for MEMS Applications. Proceedings of the 2015 IEEE 10th International Conference on Nano/Micro Engineered and Molecular Systems (NEMS).

[24] Lachaux J., Alcaine C., Gómez-Escoda B., Perrault C.M., Duplan D.O., Wu P.-Y.J., Ochoa I., Fernandez L., Mercier O., Coudreuse D. (2017). Thermoplastic elastomer with advanced hydrophilization and bonding performances for rapid (30 s) and easy molding of microfluidic devices. Lab Chip.

[25] Lachaux J., Salmon H., Loisel F., Arouche N., Ochoa I., Fernandez L.L., Uzan G., Mercier O., Veres T., Roy E. (2019). Soft Thermoplastic Elastomer for Easy and Rapid Spin-Coating Fabrication of Microfluidic Devices with High Hydrophilization and Bonding Performances. Adv. Mater. Technol..

[26] Zhuravlev E., Schick C. (2010). Fast scanning power compensated differential scanning nano-calorimeter: 2. Heat capacity analysis. Thermochim. Acta.

[27] Wang B., Lai J., Li H., Hu H., Chen S. (2013). Nanostructured vanadium oxide thin film with high TCR at room temperature for microbolometer. Infrared Phys. Technol..

[28] Vandooren A.A., Muller B.W. (1981). Influence of Experimental-Variables on Curves in Differential Scanning Calorimetry. 2. Effects on Baseline-Related Characteristics. Thermochim. Acta.

[29] Donovan J.W., Mapes C.J., Davis J.G., Garibaldi J.A. (1974). A Differential Scanning Calorimetric Study of the Stability of Egg White to Heat Denaturation. J. Sci. Food Agric..

[30] Clark C.A., Schwinefus J.J., Schaefle N.J., Muth G.W., Miessler G.L. (2008). Lysozyme thermal denaturation and self-interaction: Four integrated thermodynamic experiments for the physical chemistry laboratory. J. Chem. Educ..

[31] Ionescu R.M., Vlasak J., Price C., Kirchmeier M. (2008). Contribution of variable domains to the stability of humanized IgG1 monoclonal antibodies. J. Pharm. Sci..

[32] Dimasi N., Gao C., Fleming R., Woods R.M., Yao X.-T., Shirinian L., Kiener P.A., Wu H. (2009). The Design and Characterization of Oligospecific Antibodies for Simultaneous Targeting of Multiple Disease Mediators. J. Mol. Biol..

[33] Weiss T., Igel G., Urban G. (2007). Chip-based scanning nano-calorimeter for protein stability analysis in biosensor membranes. Proceedings of the Transducers ’07 & Eurosensors XXI, Lyon, France, 10–14 June 2007.

