# Supplementary material for: A High-Throughput MEMS-Based Differential Scanning Calorimeter for Direct Thermal Characterization of Antibodies
Source: Biosensors (Basel). 2022 Jun 16;12(6):422. doi: 10.3390/bios12060422 (PMC9221040; doi:10.3390/bios12060422)
Supplement: Supplementary file 1 [file biosensors-12-00422-s001.zip › biosensors-1746328-supplementary.pdf]

The fabrication process of the MEMS DSC device started by spin-coating PI2611 (HD MicroSystems) on a silicon wafer. After curing, the polyimide substrate was formed with a thickness of 10 µm. (Figure S1a). Then, a layer of VO_x (100 nm) was deposited on the polyimide substrate by DC sputtering (Figure S1b). During the deposition process, the Ar flow rate was 50 sccm, while the O_2 flow rate was 8 sccm. The Au/Ti layer was then patterned on the VO_x to form the thermistor by photolithography, e-beam deposition of Au/Ti (100 nm), and liftoff (Figure S1c). After that, a layer of polyimide was fabricated on the top of the thermistor as the dielectric layer (10 µm) (Figure S1d). In the next step, a layer of Au/Ti was patterned on the top of the dielectric layer by photolithography, e-beam deposition of Au/Ti (100 nm), and liftoff to form the microheater (Figure S1e). Lastly, a protective polyimide layer was fabricated on the top of the microheater (Figure S1f).

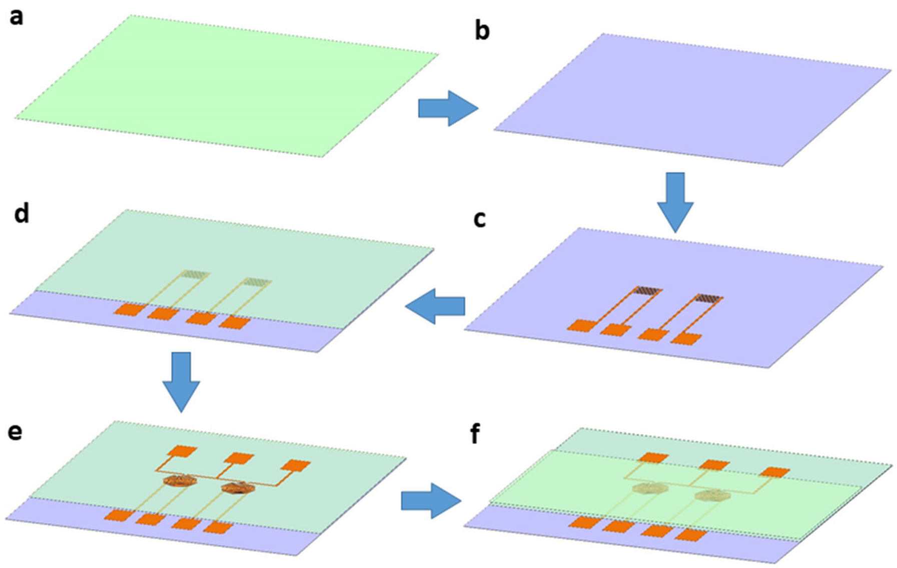

Figure S1. The fabrication process of the MEMS DSC device.

The detailed structure of the double-layer microfluidic device is shown in Figure S2. The top cavity together with the side air gap channel can significantly increase the thermal insulation of the microfluidic device, thus increasing the sensitivity of the MEMS DSC.

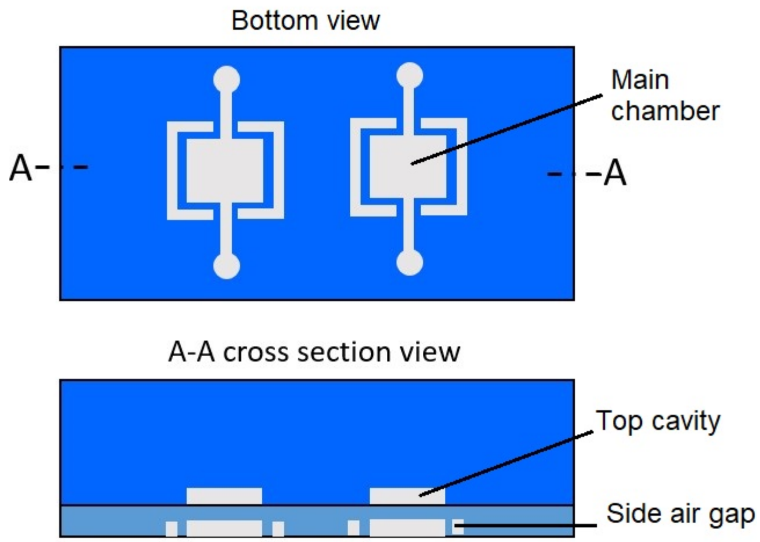

Figure S2. The structure of the microfluidic device.

Three designs of microfluidic device designs were developed to compare their thermal insulation. Design 1 consisted of two identical chambers with a volume of 0.63 µL ($1.2 \times 1.4 \times 0.25\ mm^3$) and channels which connected to the inlets and outlets for liquid delivery (Figure S3-1a). In design 2, side air gaps were added around the two chambers (Figure S3-2a). The width of the air gap was 100 µm. In design 3, a top layer with air cavity was added to the chamber layer to form the two-layer design based on design 2 (Figure S3-3a). The dimension of the top air cavity was $1.2 \times 1.4 \times 0.25\ mm^3$. An FEA simulation was carried out to compare their thermal insulation. Since the reference and sample chambers were identical, only one chamber was considered in the FEA model. The inlet, outlet, and channels were also removed to further simplify the model. In the FEA study, the chamber was filled with pure water, and a constant power of 5 mW was added to the water. The calculation time was 24 s in 80 steps. The temperature distributions of the three designs are shown in Figures S3-1b, S3-2b, and S3-3b, respectively. The environment temperature was 293.15 K (20 ℃). The temperature recordings of the centric point of the chambers in these three designs are shown in Figure S4. The thermal insulation of the microfluidic device can be expressed by the rising temperature over the input power. The results of comparing the three designs show that the side air gap could increase the thermal insulation slightly, while the top air cavity could significantly increase the thermal insulation by 1.8 times.

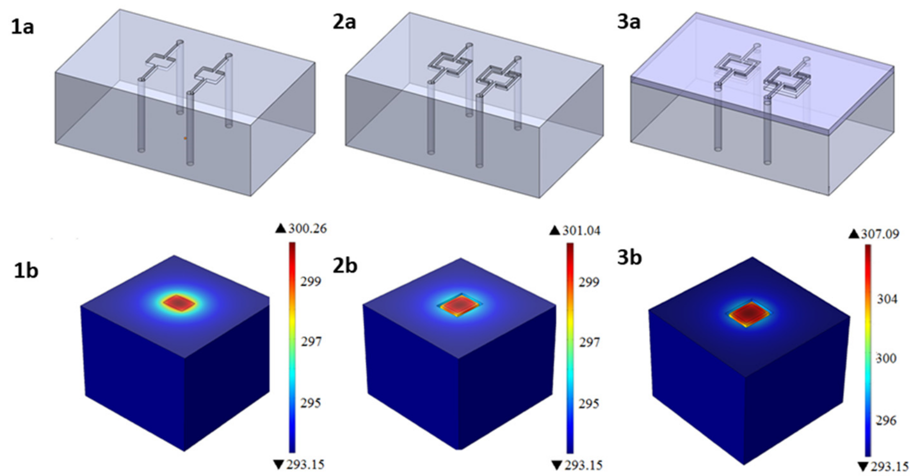

Figure S3. Three microfluidic device designs and the FEA simulation results of their step response.

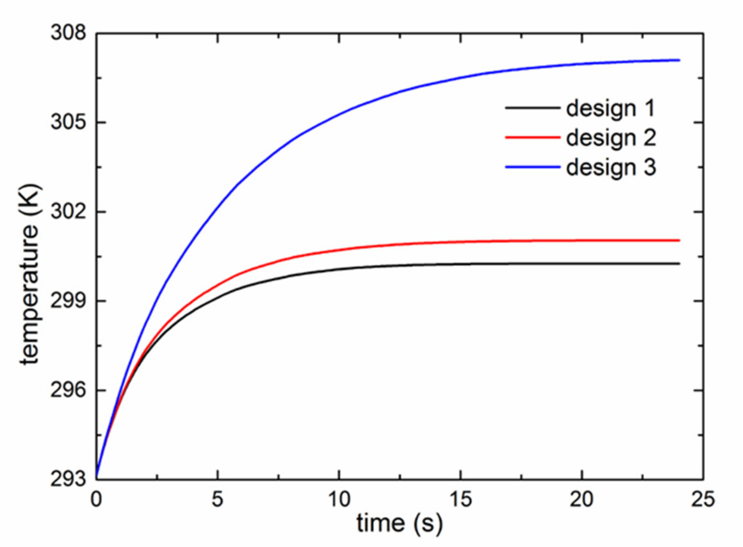

Figure S4. Step response of the three microfluidic device designs.

Figure S5 shows the schematic diagram of the MEMS DSC measurement system. The MEMS DSC was placed in a temperature module. The temperature of the module was precisely controlled by a feedback temperature controller. The two thermistors together with two external decade resistor boxes formed the Wheatstone bridge for differential power sensing. The source meter provided the input voltage for the bridge and was also used for sensitivity calibration. LabVIEW was used for temperature control and output recording.

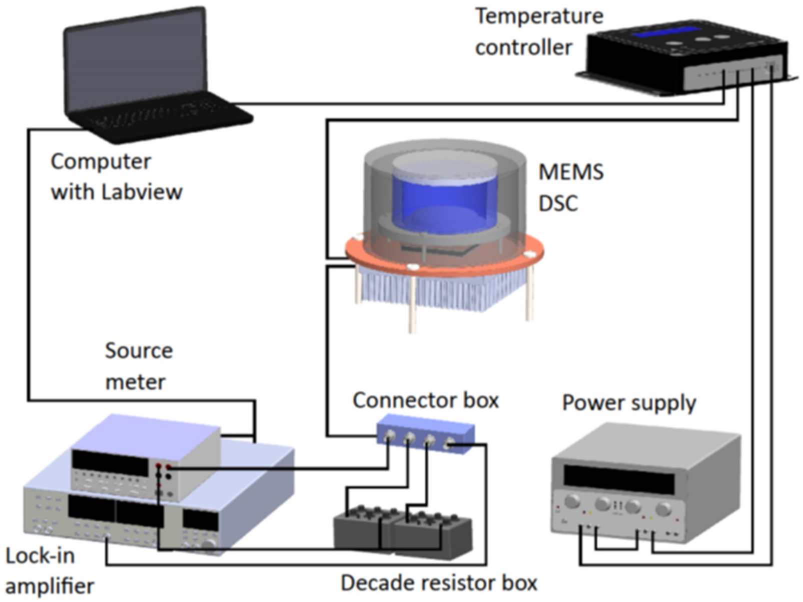

Figure S5. The schematic diagram of the MEMS DSC measurement system.

The relationship between the thermistor resistance and temperature is shown in Figure S5. VO_x is a semiconductor material, whose resistance can be expressed by Equation (S1).

$$R = R_0 e^{\frac{E_a}{kT}}, \tag{S1}$$

where R_0 is a constant, E_a is the activation energy determined by the band gap, k is the Boltzmann constant, and T is the temperature in kelvin. The TCR (α) can be expressed by Equation (S2).

$$\alpha = \frac{E_a}{kT^2}. \tag{S2}$$

The activation energy for VO_x was 0.197 eV and the TCR was −2.51% (300 K). Equation (S2) suggests that the TCR of VO_x decreases as the temperature increases. The characterization of the VO_x thermistor is shown in Figure S6.

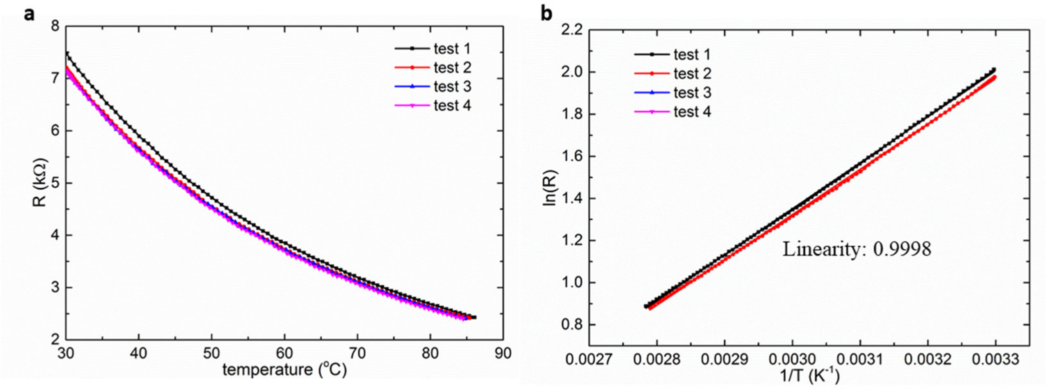

Figure S6. Calibration of the VO_x-based thermistor.

The linearity of the temperature scanning was calibrated using a commercial temperature sensor (MP-3189) for real-time temperature recording, achieving a high linearity (*R*-squared of 0.99917) at a scanning rate of 45 °C/min.

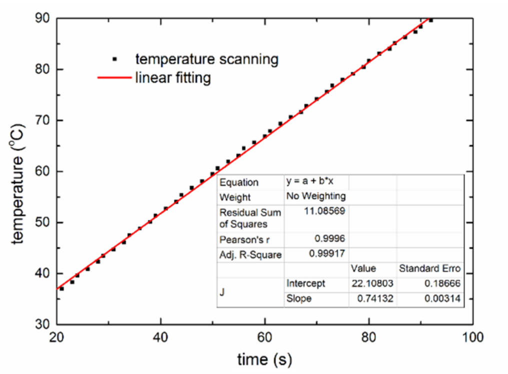

Figure S7. Heating calibration of the MEMS DSC.

The DSC measurement system achieved high stability when in equilibrium. The temperature fluctuation was within 40 μK (Figure S8).

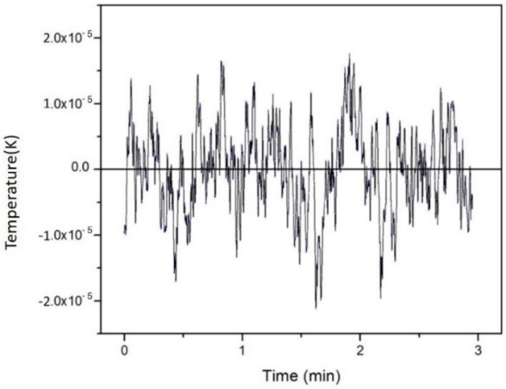

Figure S8. Temperature fluctuation of the MEMS DSC when in equilibrium.

In the sensitivity measurement, different input powers were loaded to the sample. Figure S9a shows the response of the MEMS DSC to the input power. As the input power increased from 5 μW to 30 μW, the response varied from 29.5 μV to 180.2 μV. The linear fitting of the input power to the voltage response is shown in Figure S9b. The coefficient of determination was 0.9992. The slope was 6.1 μV/μW. Five devices were used to evaluate the consistency of the MEMS DSC.

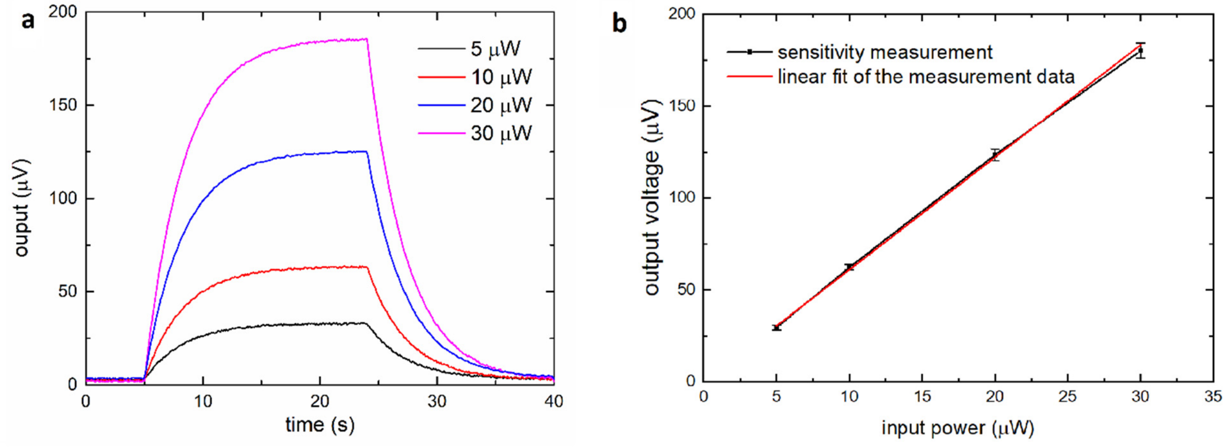

Figure S9. (a) Step response of the MEMS DSC to different input powers. (b) Linear fit of the output voltage to the input power.

The service life of the MEMS-based DSC was further studied. At such a high scanning rate (45 °C/min), the denatured sample can easily stick on the wall of the polymer chamber after chamber cleaning. The protein residue can influence the accuracy of the test result and, hence, shorten the service life of the DSC. Figure S10 shows the lysozyme test results using the same MEMS DSC device. They indicate that the device can be only used two or three times, revealing its poor repeatability and further suggesting it for disposable use.

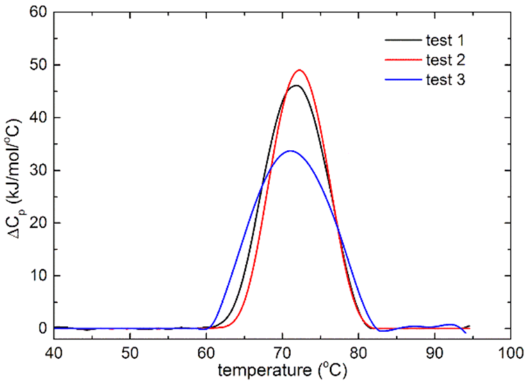

Figure S10. Repeatability test of the DSC device.

The mAb consists of two identical heavy chains and two identical light chains. Each chain folds into different domains. There are two domains in the light chain, constant light (CL) and variable light (VL), and four domains in the heavy chain, variable heavy (VH) and constant heavy (CH_1, CH_2, and CH_3). Papain digestion of mAb can divide the molecule into two fragments: the Fab fragment and the fragment of crystallizable region (Fc fragment). In one particular novel format of bispecific antibodies, two pairs of variable regions are linked with a specific amino-acid sequence to create an enhanced Fab with outer and inner variable domains (DVD-Ig). As a result, the DVD-Ig is capable of simultaneously binding two separate antigens that are related to a particular disease state. Compared with the mAb, the DVD-Ig with bispecific activities has the potential to improve clinical efficacy through simultaneous targeting of dual antigens and provides a more extensive modification of biochemical pathways related to a particular disorder [5]. The molecular structures of the Fab, mAb, and DVD Ig are shown in Figure S11. The surface charge distributions of the three proteins are shown in Figure S12.

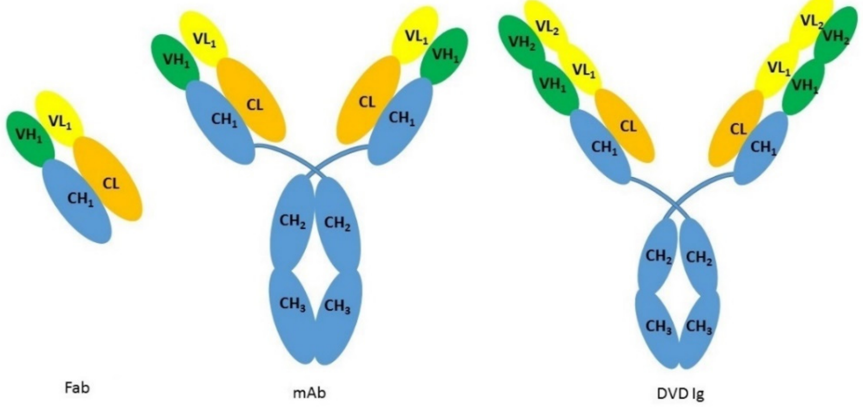

Figure S11. The molecular structure of the Fab, mAb, and DVD Ig.

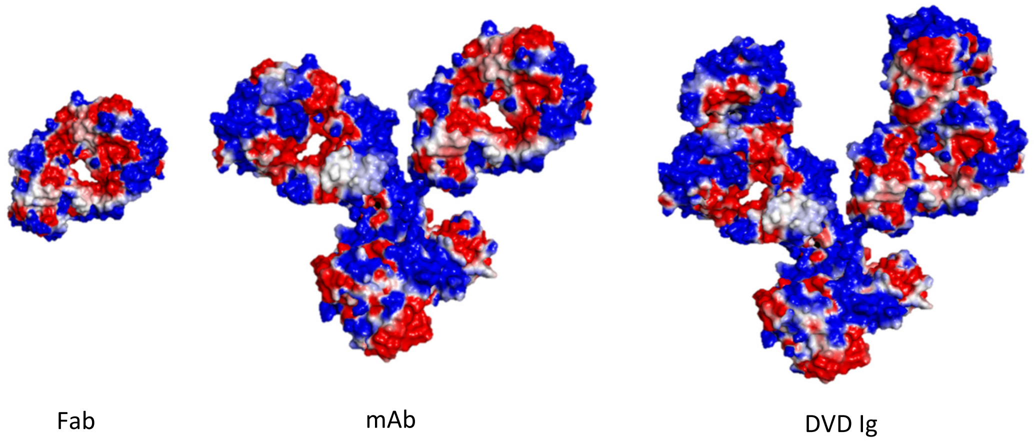

Figure S12. Surface charge distribution of the Fab, mAb, and DVD Ig. The surface electrostatic potential was calculated using the PyMOL molecular graphics program (white, neutral; blue, positive charge; red, negative charge).
